# Wheat straw biochar-supported nanoscale zerovalent iron for removal of trichloroethylene from groundwater

**DOI:** 10.1371/journal.pone.0172337

**Published:** 2017-03-06

**Authors:** Hui Li, Ya Qin Chen, Shuai Chen, Xiao Li Wang, Shu Guo, Yue Feng Qiu, Yong Di Liu, Xiao Li Duan, Yun Jiang Yu

**Affiliations:** 1 State Environmental Protection Key Laboratory of Environmental Risk Assessment and Control on Chemical Process, School of Resources and Environmental Engineering, East China University of Science and Technology, Shanghai, P.R. China; 2 School of Bioengineering, East China University of Science and Technology, Shanghai, P.R. China; 3 Center of Environmental Health Research, South China Institute of Environmental Sciences, Guangzhou, P.R. China; 4 School of Energy and Environmental Engineering, University of Science and Technology Beijing, Beijing, China; RMIT University, AUSTRALIA

## Abstract

This study synthesized the wheat straw biochar-supported nanoscale zerovalent iron (BC-nZVI) via in-situ reduction with NaBH_4_ and biochar pyrolyzed at 600°C. Wheat straw biochar, as a carrier, significantly enhanced the removal of trichloroethylene (TCE) by nZVI. The pseudo-first-order rate constant of TCE removal by BC-nZVI (1.079 h^−1^) within 260 min was 1.4 times higher and 539.5 times higher than that of biochar and nZVI, respectively. TCE was 79% dechlorinated by BC-nZVI within 15 h, but only 11% dechlorinated by unsupported nZVI, and no TCE dechlorination occurred with unmodified biochar. Weakly acidic solution (pH 5.7–6.8) significantly enhanced the dechlorination of TCE. Chloride enhanced the removal of TCE, while SO_4_^2−^, HCO_3_^−^ and NO_3_^−^ all inhibited it. Humic acid (HA) inhibited BC-nZVI reactivity, but the inhibition decreased slightly as the concentration of HA increased from 40 mg∙L^-1^ to 80 mg∙L^-1^, which was due to the electron shutting by HA aggregates. Results suggest that BC-nZVI was promising for remediation of TCE contaminated groundwater.

## Introduction

Trichloroethylene is a widespread and persistent contaminant of groundwater which poses a serious threat to groundwater environmental safety and human health[1]. Removal of TCE from groundwater is a challenging task considering the complex subsurface environment. Zerovalent iron (ZVI) has been effectively used to degrade halogenated organic compounds since 1994[2]. Recently, ZVI research has shifted to the nanoscale material[3], because ZVI is easily passivated and has a relatively slow reaction rate due to its large size[4]. In contrast, nanoscale zerovalent iron (nZVI) has high dechlorination rates and transform chlorinated solvents completely without accumulation of chlorinated byproducts[5,6]. However, there are still many factors that limit the application of nZVI, such as poor stability, poor mobility and potential ecotoxicity[7].

The biggest challenge facing the use of nZVI is its tendency to agglomerate, due to its high surface energy and magnetic interaction[8], which severely limits its stability and mobility in groundwater environments[9].

The main approaches for reducing nZVI aggregation include coating with organic polymer materials, including guar gum[10], carboxyl methyl cellulose (CMC)[11] and polyacrylic acid (PAA)[12], and inorganic adsorbent material, including sepiolite[13], smectite[14], alginate bead[15] and activated carbon[9,16]. Activated carbon materials can not only effectively decrease nZVI aggregation, but also rapidly increase the concentrations of contaminants in the micro environments surrounding nZVI because of its adsorption capacity[17]. Although activated carbon immobilizes nZVI and improves the TCE removal efficiency, its preparation consumes a lot of energy. An environmentally friendly support for nZVI with high adsorption capacity at a low cost would be highly desirable.

Biochar, a charcoal produced by heating biomass under anoxic conditions, attracts growing interest as a promising and environmentally-friendly support and adsorbent material[18]. Biochar has a porous structure and a large specific surface area and also possesses large numbers of oxygen-containing functional groups that are formed during the pyrolysis process. These characteristics suggest biochar may be used as an alternative to activated carbon to remove organic contaminants and heavy metals from fluids, such as Cu, pesticides and chlorophenol[19–22]. Recently, biochar produced from soybean stover, peanut shells and pine needles at various temperatures has been used to remove TCE from aqueous solutions[23,24]. The raw biomass and pyrolysis temperature significantly affect TCE adsorption[23]. On the other hand, biochar has also been used to immobilize (i.e., to disperse and stabilize) nanoparticles[25–27]. For example, Yan et al.[25] prepared nZVI supported by rice hull biochar and used it as a persulfate activator to enhance the removal of TCE from aqueous solutions. Devi et al.[28,29] also prepared two magnetic biochar composites, one with nZVI and the other with Ni-ZVI for efficiently adsorbing and dechlorinating pentachlorophenol in effluent. However, further studies of nZVI supported by different types of biochar are required to determine the ability to remove TCE in the contaminated groundwater.

In this study, the adsorption and dechlorination of TCE by biochar-supported nZVI (BC-nZVI) were investigated in comparison with that of the activated carbon supported nZVI (AC-nZVI). The effect factors on reactivity of the BC-nZVI composites were explored, including pH of solution, the presence of common anions and humic acids.

## Materials and methods

### Materials and chemicals

Wheat straw was purchased from a farmer in Lianyungang, Jiangsu Province, China. Commercial available activated carbon was obtained from the Shxh Carbon Corporation (Shanghai, China). Deionized water was obtained from East China University of Science and Technology (Shanghai, China). The oxygen was removed by purging with pure nitrogen gas. Ethanol (99.7%), ferrous sulfate heptahydrate (99.0%), sodium borohydride (96%), *n*-hexane (97.0%), hydroxylamine hydrochloride (98.5%), sodium acetate (99.0%), TCE (99.0%), sodium chloride (99.5%), sodium sulfate (99.0%), sodium nitrate (99.0%) and sodium bicarbonate (99.0%) were obtained from Lingfeng Chemical Reagent Co. Ltd. (Shanghai, China). Phenanthroline (99%) was obtained from Huzhen Chemical Technology Co., Ltd. (Shanghai, China). Sodium hydroxide (96%) and hydrochloric acid (30%) were obtained from Tianlian Chemical Technology Co., Ltd. (Shanghai, China). Fulvic acid (≥90%) was obtained from Bailingwei Chemical Technology Co., Ltd. (Shanghai, China). All chemicals were analytical grade.

### Preparation of the BC-nZVI composite

Biochar was produced by pyrolyzing fresh wheat straw at 600°C. Briefly, wheat straw was ground and sieved to give a powder with particles with diameters<1.0 mm. The powdered material was pyrolyzed at 600°C for 2 h in a tube furnace with a nitrogen atmosphere. The heating rate was 5°C∙min^-1^. Once the temperature reached 600°C, the temperature was maintained for 2 h to allow complete carbonization to occur. The biochar was cooled, treated with 1 M HCl for 12 h, and washed three times with deionized water (200 mL per 1 g biochar) to remove impurities. The cleaned biochar was dried at 75°C and stored in a drying chamber containing silica gel.

The nZVI particles were prepared by reducing FeSO_4_·7H_2_O with NaBH_4_, following a procedure similar to Su et al.[30]. Critical conditions include excess NaBH_4_ to ensure thorough reduction of Fe^2+^ and 2:3 ratio of water to ethanol to ensure uniform nanoparticles. BC-nZVI was prepared by suspending 1.5 g biochar in 100 mL of 0.25 M FeSO_4_·7H_2_0 in 2:3 (v/v) water:ethanol in a tree-neck flask. The solution was stirred at 700 rpm. Particles of nZVI were deposited on biochar surfaces and in pores by adding, dropwise (2 drops∙s^-1^), 100 mL of 0.55 M NaBH_4_ (pH 11) with vigorous stirring. The mixture was stirred an additional 30 min. The resultant BC-nZVI was washed with deoxygenated deionized water three times to remove inorganic ions and with ethanol to remove the water, and stored in an anaerobic chamber. The whole process was carried out under a nitrogen atmosphere. The mass ratio of nZVI to biochar was approximately 1:1. The AC-nZVI was prepared in the same way.

### TCE removal kinetics

TCE removal experiments were conducted in a 150 rpm incubator shaker at room temperature (20±1°C) in the dark. BC-nZVI, BC, AC-nZVI and AC were added (0.1 g∙L^-1^) to 100 mL serum bottles containing 100 mL aqueous solution including 30 mL TCE (pH 7.0). Each bottle was immediately sealed with a polytetrafluoroethylene stopper and an aluminum cap. Sample was collected using a glass syringe at each specified time interval and immediately passed through a 0.22 mm membrane filter. The filtered sample was analyzed by gas chromatography to determine the TCE concentration. TCE removal experiment was performed in triplicate to determine the reproducibility.

The TCE removal efficiency was determined:

$$\text{TCE removal efficiency }\left(\%\right)\;=\;\frac{C_{0,TCE}-C_{t,TCE}}{C_{0,TCE}}\times 100$$ (1)

Because 1 mol of TCE contains 3 mol of Cl^−^, the degradation efficiency of TCE was calculated:

$$\text{TCE dechlorination efficiency }\left(\%\right)\;=\;\frac{C_{0,TCE}-C_{t,Cl^{-}}\times 1.234}{C_{0,TCE}}\times 100$$ (2)

In Eqs (1) and (2), C_0,TCE_ (mg∙L^-1^) was the initial concentration of TCE in the solution. C_t,TCE_ (mg∙L^-1^) was the TCE concentration in the solution at time t. C_t,Cl_^−^ (mg∙L^-1^) was the Cl^−^ concentration in the solution at time t. C_t,Cl_^−^×1.234 was determined from the equation $\frac{C_{t,Cl^{-}}}{3\times M_{Cl^{-}}}\times M_{TCE}$ and the term “C_t,Cl_^−^×1.234” was the concentration of dechlorinated TCE (assuming 3 mol Cl^-^ was liberated for each mole of TCE dechlorinated)[31].

TCE removal kinetics followed a pseudo-first-order rate model and the rate constant was determined:

$$\frac{dC_{t,TCE}}{dt}=-k_{obs,TCE}C_{t,TCE}$$ (3)

$$\text{ln}(\frac{C_{0,TCE}}{C_{t,TCE}})=k_{obs,TCE}t$$ (4)

where C_0_ (mg∙L^-1^) was the initial TCE concentration in the solution. C_t,TCE_ (mg∙L^-1^) was the TCE concentration in the solution at time t. k_obs,TCE_ (h^-1^) was the removal rate constant and t was the reaction time.

### TCE determination

For analysis of aqueous TCE, a 0.5 mL sample was added in a 5 mL brown bottle containing 1.5 mL *n*-hexane. Then the bottle was placed on a vortex shaker at 2000 rpm for 3 min. For analysis of TCE adsorbed on the composite, the residual material was obtained by filtering the solution and immediately placed in 5 mL brown bottles containing 1.5 mL *n*-hexane and extracted for 5 min on a vortex shaker[31]. The TCE concentrations in the *n*-hexane extracts were determined by an Agilent 7890 gas chromatograph (Agilent Technologies, Santa Clara, CA, USA) equipped with an electron capture detector and a DB-VRX capillary column (60 m×0.25 mm×1.4 μm film thickness; Agilent Technologies). Samples were injected at a 20:1 split ratio. Respective temperatures of the injection port and detector were 260°C and 240°C. The carrier gas was ultrapure nitrogen and the flow rate was 20 mL∙min^-1^. The temperature was programmed to increase from 45°C to 190°C at 12°C∙min^-1^, which was maintained for 2 min.

### Analysis methods

Surface area, total pore volume and pore diameter of BC-nZVI were measured using a Brunauer-Emmett-Teller (BET) analyzer (ASAP 2010; Micrometrics, Norcross, GA, USA). Surface morphology and surface elemental compositions of BC-nZVI were determined using a field emission-scanning electron microscope (S-3400N; Hitachi High-Technologies Corporation, Tokyo, Japan). The crystal structures and crystallinity of BC-nZVI before and after reaction were characterized by X-ray diffraction (D/MAX-2550 VB/PC; Rigaku, Tokyo, Japan) with a 10°to 80° scanning range. Surface functional groups were determined by Fourier transform infrared (FT-IR) spectrometry.

Concentrations of Fe^2+^ and Fe^3+^ in the aqueous phase were determined by UV spectrophotometer (UV1800; Shimadzu, Kyoto, Japan), using the phenanthroline method[32]. The Cl^−^ concentrations in the aqueous phase were determined by ion chromatography using an ICS 1000 system (Thermo Fisher Scientific, Waltham, MA, USA). The pH was measured with a pH electrode.

### Statistical analysis

Statistical analysis was performed using SPSS 20.0 software. An analysis of the data using analysis of variance (ANOVA) with between- and within-subject factors was conducted for each experiment. A repeated-measures ANOVA was conducted to analyze the TCE removal kinetics, with Time (0, 5, 15, 30, 60, 90, 160, 210 and 260 min) as the within-subjects factor and Group (control, nZVI, AC, BC, AC-nZVI and BC-nZVI) as the between-subjects factor. A repeated-measures ANOVA was conducted to analyze the effect of pH on TCE removal, with Time (0, 5, 15, 30, 60, 90 and 160 min) as the within-subjects factor and pH (4.4, 5.7, 6.8 and 9.8) as the between-subjects factor. A repeated-measures ANOVA was conducted to analyze the effect of humic acid on TCE removal, with Time (0, 5, 15, 30, 60 and 90 min) as the within-subjects factor and HA concentration (0, 1, 5, 10, 40 and 80 mg∙L^-1^) as the between-subjects factor. We further analyzed significant main effects and interactions (*p*<0.05) in the factorial ANOVAs using Least Significant Difference post hoc tests.

## Results and discussion

### Characterization of the BC-nZVI

The specific surface areas of BC-nZVI and AC-nZVI were smaller than those of biochar and activated carbon (Table 1). This was attributed to loaded nZVI which had a much lower surface area than BC or AC. Pore volumes of BC-nZVI and AC-nZVI also were smaller than those of BC or AC. The average pore diameter of BC-nZVI was sufficient for entry of TCE contaminants.

**Table 1:** BET surface areas and pore volumes of the nZVI, biochar, activated carbon, BC-nZVI, and AC-nZVI.

Samples	BET surface area	Total pore volume	Average pore diameter
	(m^2^∙g^-1^)	(cm^3^∙g^-1^)	(nm)
nZVI	12.34	0.04	-
biochar	155.7	0.12	8.4
Activated carbon	1416.4	0.93	2.6
BC-nZVI	137.2	0.11	8.0
AC-nZVI	1002.7	0.90	4.1

The surface morphologies of the biochar, fresh BC-nZVI and exhausted BC-nZV are shown in Figure A in S1 File. It showed that the wheat straw biochar had a rough and porous surface (Figure A1 in S1 File), which provided the suitable sites to support nZVI. The nZVI particles that were immobilized on the biochar were well dispersed (Figure A2 in S1 File) and the biochar became a large sheet-like structure as a result of infiltration of water and stirring vigorously during preparation process. The nZVI particles dispersed on the biochar were hard to distinguish after reacting with TCE (Figure A3 in S1 File), because nZVI particles were oxidized during the reaction.

**Fig 1:**
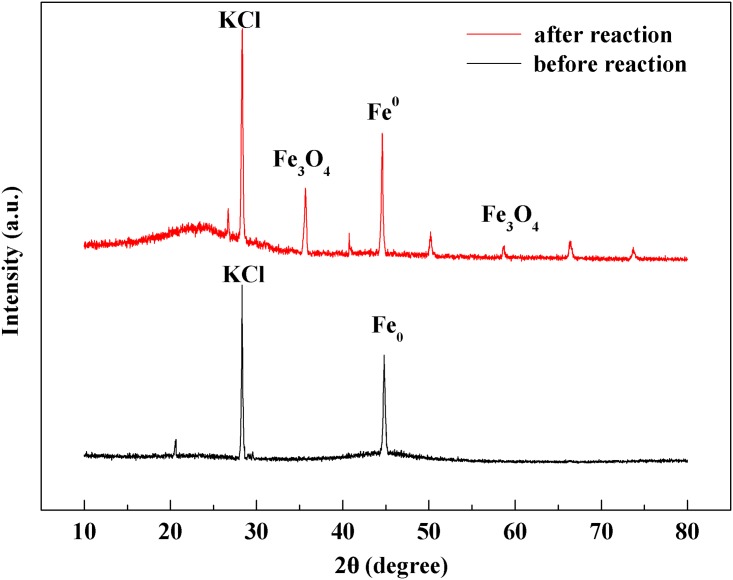
X-ray diffractograms for BC-nZVI before and after reaction.

The major XRD peak at 2θ = 44.7° in the XRD pattern of the fresh BC-nZVI (Fig 1) confirmed that ZVI had formed on the biochar surfaces[29]. Significant peaks at 2θ = 35° and 2θ = 57° appeared after the reaction with TCE, indicating the formation of magnetite (Fe_3_O_4_) on the nZVI surfaces. The peak at 2θ = 28.7° was due to KCl contained in the biochar.

The FT-IR bands between 1500 cm^-1^ and 1700 cm^-1^ were attributed to C = C and C = O stretch vibrations (Figure B in S1 File). After the reaction with TCE, the sizes of the C = C and C = O peaks decreased, because TCE was adsorbed to the pore in BC-nZVI particles.

### TCE removal kinetics

**Fig 2:**
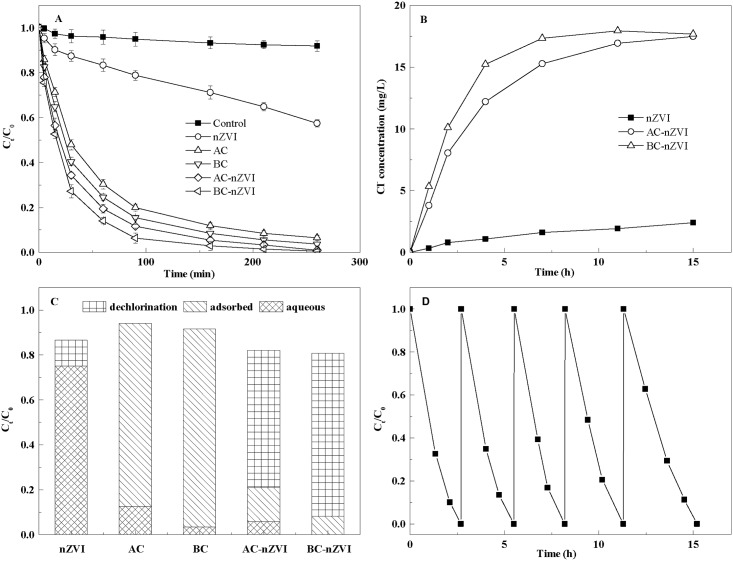
Removal of TCE by the BC-nZVI composite. **A**, TCE removal kinetics by BC-nZVI, AC-nZVI, nZVI, BC and AC; **B**, Changes in the Cl^−^ concentration during the TCE removal process; **C**, TCE mass balance taking into account sorbed, aqueous phase and dechlorinated TCE after 15 h; **D**, Removal of TCE by the BC-nZVI in the successive treatments.

More than 90% of TCE was removed within 200 min by BC-nZVI, AC-nZVI, BC and AC, while only about 55% of TCE was degraded by bare nZVI (Fig 2). Pseudo-first-order rate constants for TCE removal (k_obs,TCE_) were: BC-nZVI>AC-nZVI>BC>AC>nZVI. The repeated measures ANOVA showed significant effects of Time (*F*_8,96_ = 10523.9, *P*<0.01) and Group (*F*_5,12_ = 756.1, *P*<0.01) and a significant Time × Group interaction (*F*_40,96_ = 577.8, *P*<0.01). TCE removal was enhanced by supporting nZVI due to the greater adsorption capacity of the BC than the AC seems a usual finding. Biochar had a greater adsorption capacity than AC, attributable to its abundant pore structure.

More Cl^−^ was released from TCE in the aqueous phase by BC-nZVI and AC-nZVI than nZVI alone, showing better performance for TCE dechlorination. Chloride release was maximum when BC-nZVI was used, as a result of the greater adsorption capacity of biochar. Mass balance calculations revealed that 88% and 81% of TCE were absorbed by biochar and activated carbon, respectively, within 15 h. Only 11% of the TCE was dechlorinated by nZVI, while 79% and 62% of TCE were dechlorinated by BC-nZVI and AC-nZVI, respectively. Results indicated both adsorption and dechlorination during the removal process and the BC-nZVI composite exhibited superior performance. As were shown in Table 1, the higher BET surface area of BC-nZVI and AC-nZVI than nZVI led to the higher adsorption performance of them than nZVI alone. Further, the BC-nZVI and AC-nZVI composite decreased the aggregation of nZVI, which increased the dechlorination reactivity of nZVI.

**Fig 3:**
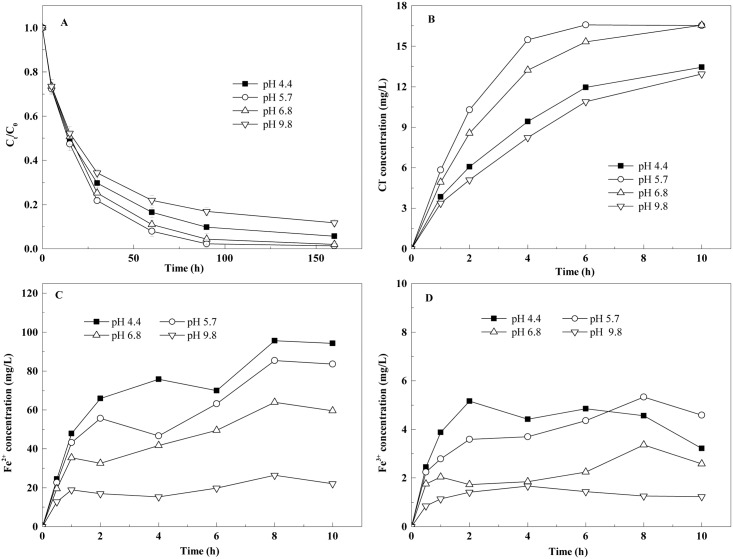
Removal of TCE by the BC-nZVI with different initial pH values. **A**, Effect of the initial solution pH on the removal of TCE by the BC-nZVI; **B**, Cl^−^ concentrations in solutions with different initial pH values; **C**, Fe^2+^ concentrations in solutions with different initial pH values; **D**, Fe^3+^ concentrations in solutions with different initial pH values.

The long-term performance of the BC-nZVI composite was evaluated by repeating TCE removal experiments in the solution. As is shown in Fig 3D, 30 mg∙L^-1^ TCE was removed by the BC-nZVI in a 2.7 h cycle. Almost 100% of the TCE can be removed even when the BC-nZVI was used in a fifth cycle, although the removal rate was slightly lower in the fourth cycle. The BC-nZVI therefore maintained its TCE removal efficiency for 15 h, indicating that the nZVI immobilized on the biochar exhibited high reactivity and stability during TCE removal.

### Effect of pH on TCE removal

TCE removal efficiency reached 90% at pH 4.4, 5.7 and 6.8, but was less efficient at pH 9.8 (Fig 3A). The highest removal rate appeared at pH 5.7, followed by the rate at pH 6.8. The rate constants (k_obs,TCE_) were in an order of pH 5.7>pH 6.8>pH 4.4>pH 9.8. The repeated-measures ANOVA showed significant effects of Time (*F*_6,48_ = 9010.8, *P*<0.01), pH (*F*_3,8_ = 10.0, *P*<0.01) and significant interaction of Time × pH (*F*_18,48_ = 14.4, *P*<0.01).

The initial pH of the solution significantly influenced the dechlorination of TCE by BC-nZVI (Fig 3B). A weakly acidic solution condition (pH 5.7–6.8) allowed TCE to be dechlorinated effectively. It was speculated that H^+^ in a weakly acidic solution reacted with the iron oxide on the nZVI surfaces, thereby providing more and more reaction sites. However, the dechlorination efficiency decreased greatly at a lower pH (<4), because a large proportion of the nZVI reacted with H_2_O[33]. Less dechlorination under highly alkaline conditions (pH 9.8) was due to the accumulation of iron oxide on nZVI surfaces, which prevented the reaction of TCE with ZVI.

Changes in the concentrations of Fe^2+^ (Fig 3C) and Fe^3+^ (Fig 3D) in the solution were examined. The Fe^2+^ concentration increased when the initial pH decreased, which was not consistent with the dechlorination of TCE. It inferred that the Fe^2+^ in aqueous phase produced not only through the dechlorination of TCE by nZVI, but also through reactions between nZVI and water. The Fe^3+^ precipitated as Fe(OH)_3_ and other iron oxides on the surface of nZVI, so the measurement of Fe^3+^ in solution was difficult because of the low solubility product constant (K_sp_) of Fe(OH)_3_ at room temperature (1.1×10^−36^).

### Effects of common inorganic anions on TCE removal

Effects of the anions (Cl^−^, SO_4_^2−^, NO_3_^−^ and HCO_3_^−^) on TCE removal by BC-nZVI were evaluated (Figure C in S1 File and Table A in S1 File). Removal rate constants increased slightly with increasing Cl^−^ concentrations. Chloride might increase dechlorination by removing the oxide coating the nZVI[34].

All of the presence of HCO_3_^−^, SO_4_^2−^, and NO_3_^−^ decreased the reactivity of BC-nZVI. The rate constants decreased in the order of control>HCO_3_^−^>SO_4_^2−^>NO_3_^−^. The rate constant was smaller in the presence of 0.5 mM HCO_3_^−^ than that without any anions, which attributed to the generation of carbonate precipitates on the surface of nZVI[35]. SO_4_^2−^ also negatively affected the BC-nZVI reactivity and the inhibition increased with concentration. Inhibition by SO_4_^2−^ likely resulted from the formation of inner-sphere complex on the nZVI surface by SO_4_^2−^[36]. NO_3_^−^ exhibited the strongest inhibitive effect with the rate constant decreased to 0.650 h^-1^ when the NO_3_^−^ concentration increased to 20 mM, which was much lower than that without any anions. Previous research has shown that NO_3_^−^ reduced by obtaining electrons from the iron surface[37], thus the competition for reactive sites might contribute to the significant inhibition of NO_3_^-^ to TCE removal by BC-nZVI.

### Effect of humic acid on TCE removal

**Fig 4:**
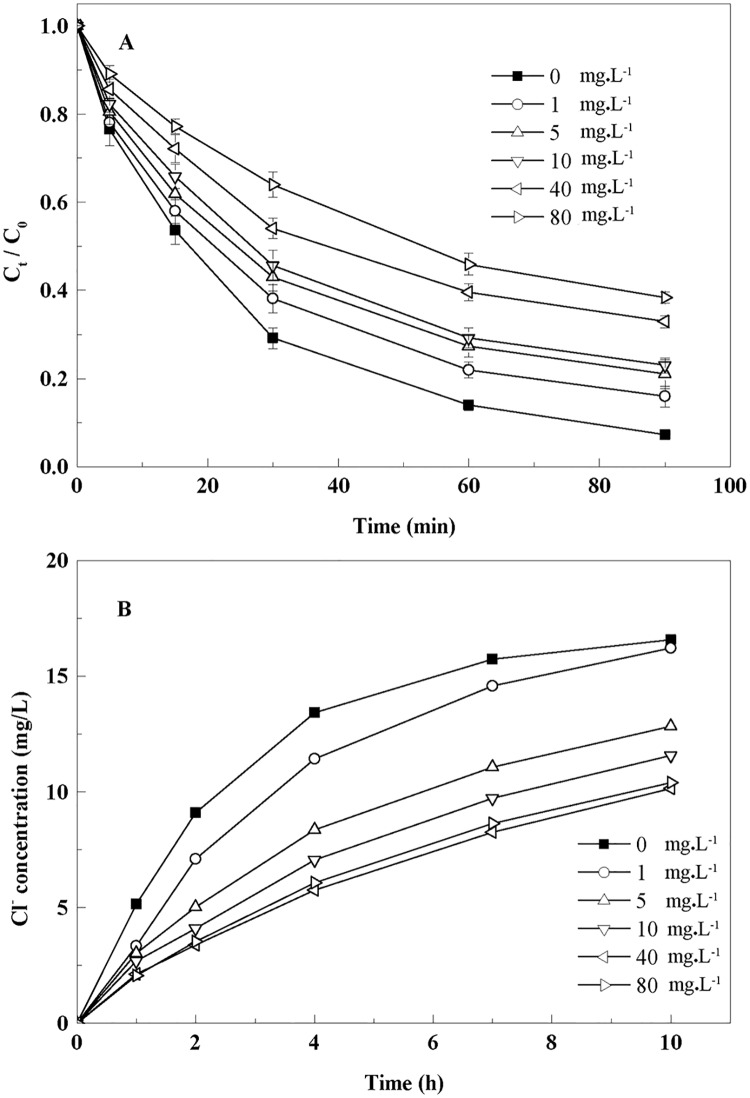
Removal of TCE by the BC-nZVI in the presence of Humic Acid (HA). **A**, Effect of HA on the removal of TCE by BC-nZVI at different HA concentrations; **B**, Cl^−^ concentrations in solutions with different HA concentrations.

Humic acid inhibited TCE removal by BC-nZVI (Fig 4A). The k_obs,TCE_ decreased with increasing HA concentration from 0–80 mg∙L^-1^. This observation was consistent with the previous study of Tseng et al. [31] that the HA competed with contaminants for reactive sites. The repeated-measures ANOVA showed significant effects of Time (*F*_5,60_ = 6522.3, *P*<0.01) and HA concentration (*F*_5,12_ = 41.9, *P*<0.01) and significant interaction of Time × HA concentration (*F*_25,60_ = 28.2, *P*<0.01).At a low HA concentration (0–1 mg∙L^-1^), TCE dechlorination was similar to that without HA (Fig 4B), while the inhibition increased significantly when the HA concentration increased from 1 to 40 mg∙L^-1^. The high concentration of the HA likely blocked the biochar pores significantly to inhibit the adsorption and dechlorination of TCE by BC-nZVI. Interestingly, the inhibition decreased when the HA concentration was increased to 80 mg∙L^-1^. Humic acid molecules tended to aggregate at high concentrations (40–80 mg∙L^-1^) and the aggregates might act as electron shuttles to enhance TCE dechlorination[38].

## Conclusions

Wheat straw biochar, an economical material, enhanced TCE removal and dechlorination by nZVI. TCE was likely removed by the BC-nZVI through a combination of adsorption by the biochar and subsequent dechlorination by the nZVI. A BC-nZVI composite was prepared that efficiently removed TCE at pH 5.7–6.8, in the presence of chloride, sulfate and bicarbonate anions, and at low HA concentrations. HA became inhibitory as concentration was increased, but HA aggregates appeared to facilitate dechlorination at the highest concentration tested. Nitrate had little effect at low concentrations but competed for electrons at higher concentrations. The mobility and lifetime of BC-nZVI should be investigated in subsurface environments and the effect on microbial activity needs to be evaluated. Because the nZVI particles could disrupt the membranes or alter membrane potential, microbial toxicity should be clear before underground injection. The present findings demonstrate the potential of the BC-nZVI for remediation of TCE-contaminated groundwater.

## Supporting information

S1 File Figure A. SEM images of the (1) biochar, (2) fresh BC-nZVI and (3) exhausted BC-nZVI. Figure B. FT-IR spectra of the biochar, fresh BC-nZVI and exhausted BC-nZVI. Figure C. Removal of TCE by the BC-nZVI in the presence of different anions. Table A. Pseudo-first-order rate constants for the removal of TCE in the presence of different anions.
(DOCX)
