# Supplementary material for: Wheat straw biochar-supported nanoscale zerovalent iron for removal of trichloroethylene from groundwater
Source: PLoS One. 2017 Mar 6;12(3):e0172337. doi: 10.1371/journal.pone.0172337 (PMC5338781; doi:10.1371/journal.pone.0172337)
Supplement: S1 File — Figure A. SEM images of the (1) biochar, (2) fresh BC-nZVI and (3) exhausted BC-nZVI. Figure B. FT-IR spectra of the biochar, fresh BC-nZVI and exhausted BC-nZVI. Figure C. Removal of TCE by the BC-nZVI in the presence of different anions. Table A. Pseudo-first-order rate constants for the removal of TCE in the presence of different anions. (DOCX) [file pone.0172337.s001.docx]

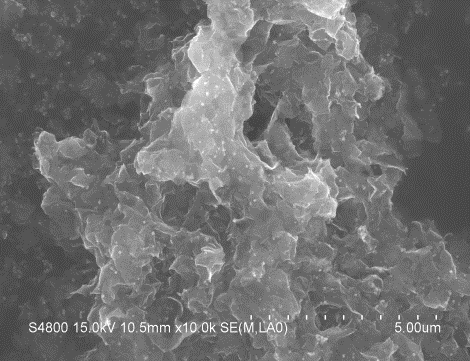


**2**


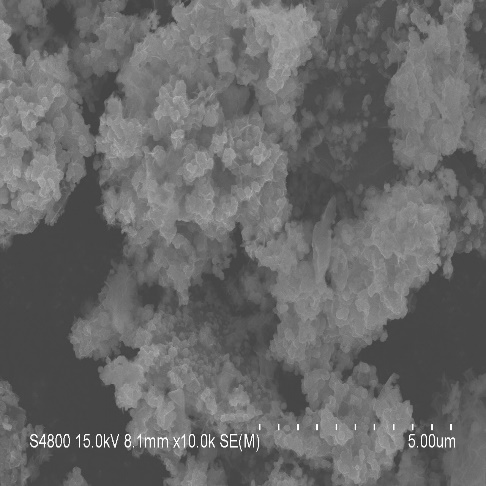


**3**


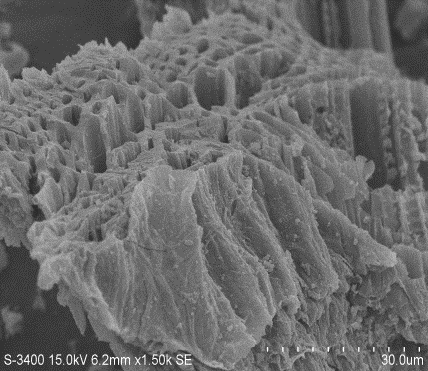


**1**

**Figure A in S1. SEM images of the (1) biochar, (2) fresh BC-nZVI, (3) exhausted BC-nZVI.**


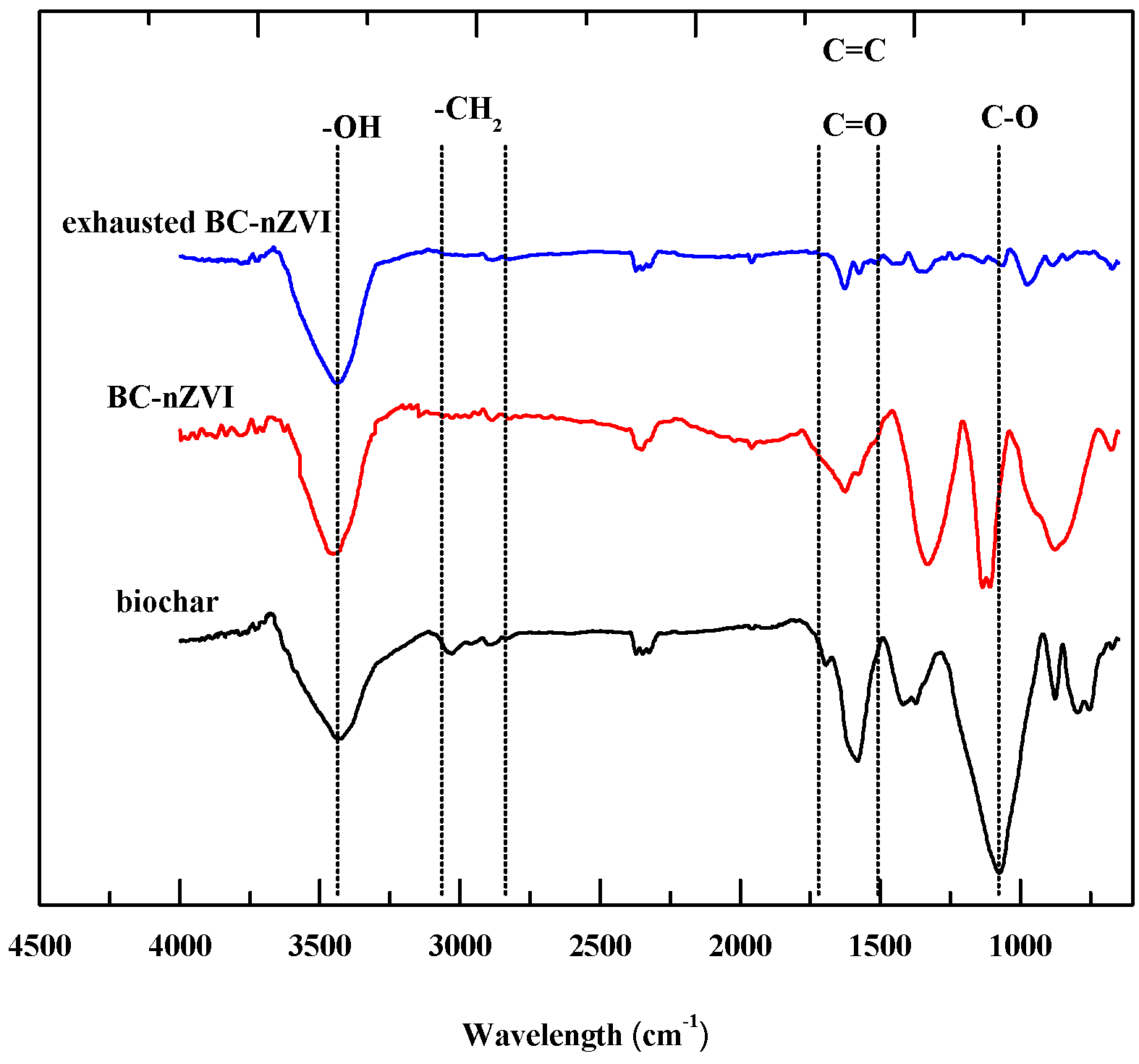


**Figure B in S1. FT-IR spectra of the biochar, fresh BC-nZVI and exhausted BC-nZVI.**


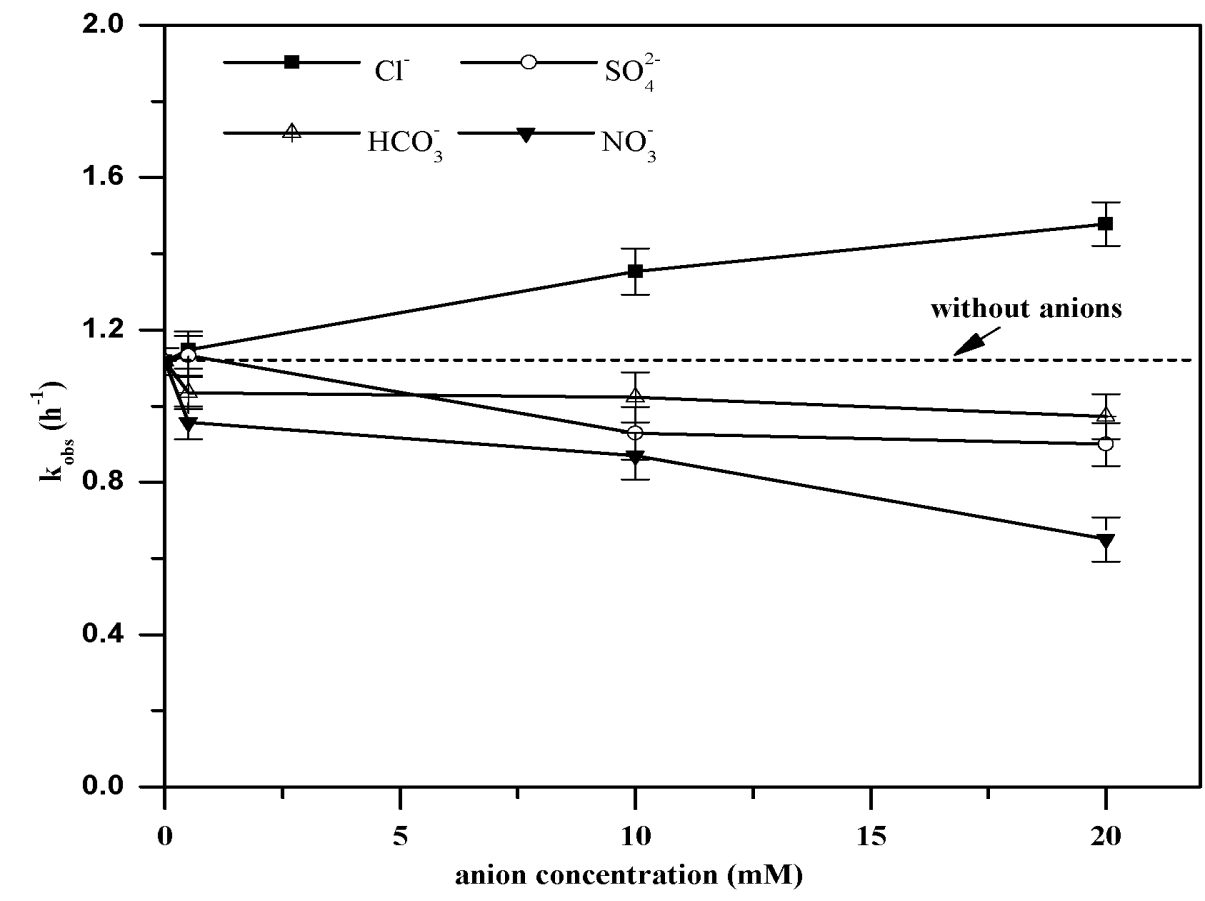


**Figure C in S1. Removal of TCE by the BC-nZVI in the presence of different anions.**

Conditions: [TCE] 30 mg∙L^-1^; BC-nZVI 1g∙L^-1^; 20±1°C.

**Table A in S1. Pseudo-first-order rate constants for the removal of TCE in the presence of different anions.**

Conditions: [TCE] 30 mg∙L^-1^; BC-nZVI dose 1g∙L^-1^; pH 7; 20±1℃.

|  | Concentrations of anions  (mM) | *k_obs_*(h^-1^) | R^2^ |
| --- | --- | --- | --- |
| Control  (without any anions) | 0 | 1.1172 | 0.9405 |
| Cl^−^ | 0.5 | 1.1473 | 0.9402 |
|  | 10 | 1.3527 | 0.9638 |
|  | 20 | 1.4775 | 0.9436 |
| SO_4_^2−^ | 0.5 | 1.1324 | 0.9622 |
|  | 10 | 0.929 | 0.9128 |
|  | 20 | 0.8993 | 0.8693 |
| NO_3_^−^ | 0.5 | 0.9565 | 0.8798 |
|  | 10 | 0.8688 | 0.8845 |
|  | 20 | 0.6496 | 0.8644 |
| HCO_3_^−^ | 0.5 | 1.0351 | 0.9421 |
|  | 10 | 1.0228 | 0.9189 |
|  | 20 | 0.9722 | 0.904 |
